# Morton’s Foot Syndrome: A Case Report and Overview

**DOI:** 10.7759/cureus.68731

**Published:** 2024-09-05

**Authors:** Akshaya Rathin Sivaji, Tony Kizhakkemuriyil Scaria, Indumathi Sundaramurthi

**Affiliations:** 1 Internal Medicine, Panimalar Medical College Hospital & Research Institute, Chennai, IND; 2 Anatomy, Panimalar Medical College Hospital & Research Institute, Chennai, IND

**Keywords:** arthrosis, distal protrusion, lengthened second metatarsal, metatarsalgia, short first metatarsal

## Abstract

Morton's foot syndrome is a hereditary condition characterized by a short first metatarsal compared to the second metatarsal. It often remains asymptomatic but can present with secondary complications. We report a case of a 46-year-old female presenting with Morton's foot syndrome, initially admitted for fatigue and weakness. Physical examination revealed characteristic foot deformities and multiple plantar calluses. This case highlights the anatomical features, pathophysiology, and biomechanical implications of Morton's foot syndrome. We discuss the challenges in differentiating between normal foot structure and Morton's foot, along with potential complications and strategies for effective management. This case report enhances understanding of Morton's foot syndrome, emphasizing the importance of recognizing this condition in clinical practice to prevent potential complications and provide appropriate management.

## Introduction

Morton's foot syndrome was first defined by Dudley Morton in 1927, who referred to this foot type as metatarsus atavicu, characterized by a short first metatarsal. He stated that in the sagittal plane, the first and second metatarsals should normally align equally. However, in Morton’s foot, the first metatarsal is positioned slightly behind the second. This results in the second metatarsal bearing more weight, leading to hypertrophy and severe pain [1]. Over time, this leads to callusing beneath the head of the second metatarsal. It is estimated to affect 4-30% of the population, but no epidemiological study has been conducted to validate this estimate [2]. Given its genetic component, most patients with Morton's foot remain asymptomatic as they often present with secondary symptoms, such as pain and a burning sensation due to excessive pressure or friction on the second metatarsal. Morton's foot syndrome is associated with several complications: increased risk of stress fractures in the second metatarsal, metatarsalgia, higher incidence of diabetic ulcers on the second metatarsal, tendency toward over-pronation, and posterior displacement of sesamoid bones in the foot [3-5].

## Case presentation

**Figure 1:**
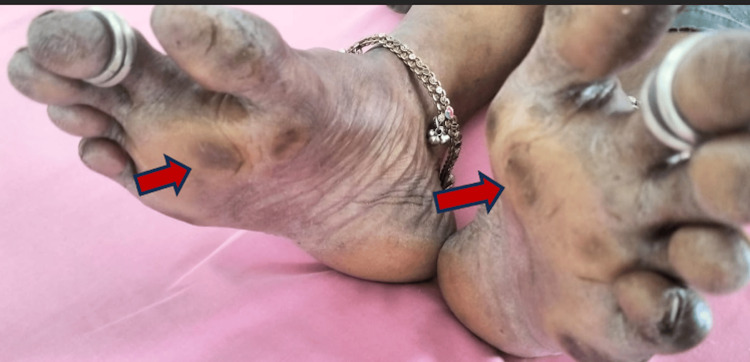
Multiple calluses in the plantar surface due to increased stress on the second metatarsal

**Figure 2:**
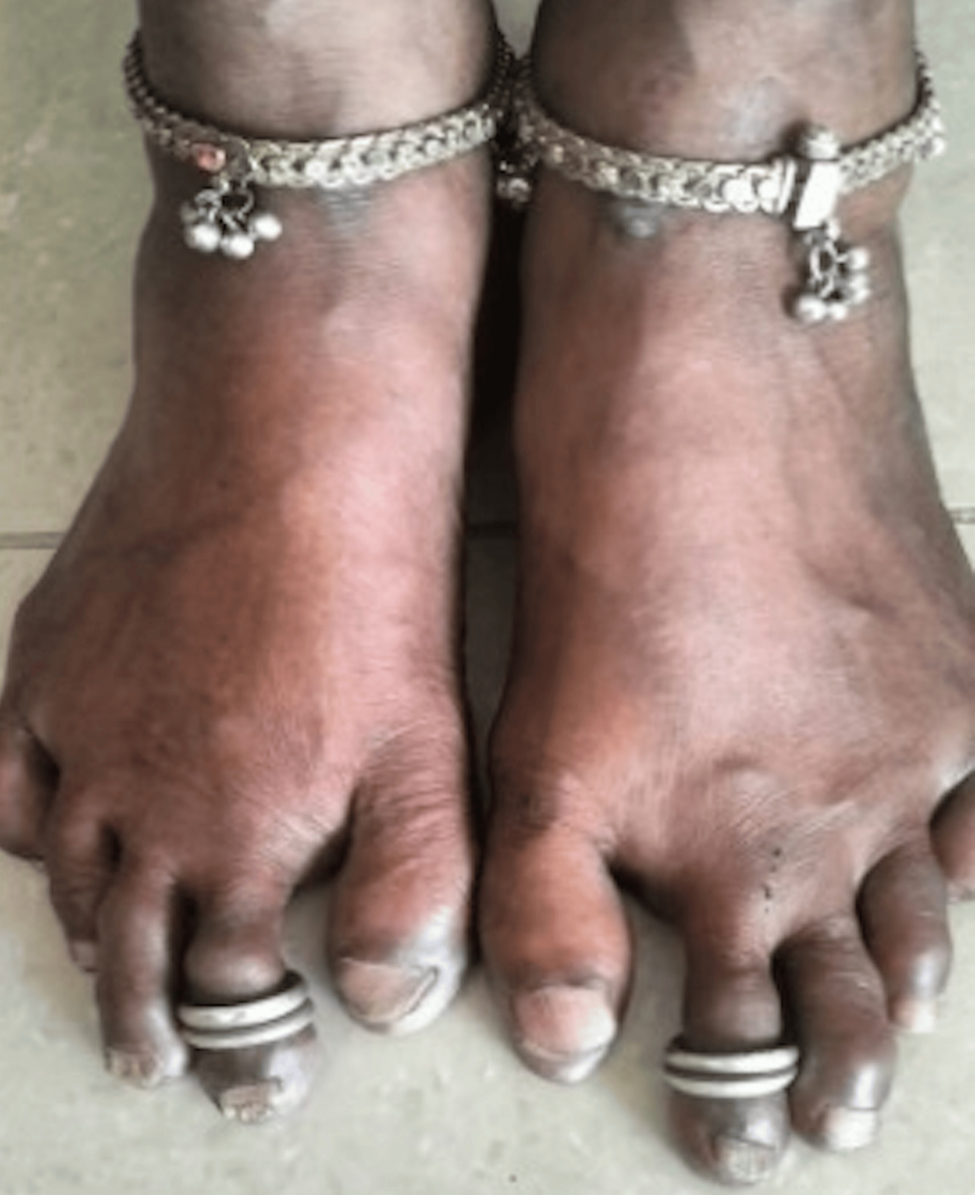
Morton's foot with long second metatarsal and short second metatarsal

A 46-year-old homemaker from Chennai, India, presented to the medicine outpatient department with excessive fatigue and a two-year history of severe pain in the sole of her foot, which has progressively altered her walking posture and increased the thickness of foot calluses. This persistent pain has significantly affected her day-to-day activities and commute impacting her quality of life, which led her to seek medical attention. In addition, she reported experiencing shortness of breath, loss of appetite, and occasional irritability at home. Initially, at the age of 17, she would occasionally feel as though something was stuck under her foot, accompanied by intermittent pain in her sole. However, this discomfort has intensified into burning pain and more intense in recent years. She has no past medical history of any allergy, diabetes mellitus, systemic hypertension, seizure disorder, or asthma. She is currently not taking any medications or any form of native treatment. On general examination, she was pale and not jaundiced and had no lymphadenopathy. Vital signs were within normal limits. On palpation, there was no presence of any organomegaly. On auscultation, she had a hemic murmur (+). Local examination revealed a shortened first metatarsal and elongated second metatarsal with multiple thick dark brown hardened calluses at the level of the metatarsal heads in both feet (Figures 1, 2).

Upon further discussion, she revealed that two of her siblings and her daughter share a similar foot structure, although none of them experienced significant pain as she does. A dermatology consultation was obtained for her calluses, and a topical keratolytic containing 0.05% clobetasol and 6% salicylic acid for the calluses was prescribed. After further consultation with the orthopedic team, she was advised to use a Morton extension pad and switch to comfortable footwear. Routine blood tests also revealed a hemoglobin of 4.8 g/dL and a mean corpuscular volume (MCV) of 42/fL. The peripheral blood smear indicated microcytic and hypochromic red blood cells, which was strongly suggestive of iron deficiency anemia. Consequently, two units of packed red blood cells were transfused, and intravenous iron replacement therapy was initiated on alternate days. However, anemia was found incidentally, and there is no significant association between both conditions. The patient granted consent for the utilization of images of her foot for study purposes. We have assured her that every effort will be taken to maintain confidentiality by refraining from disclosing name, identity, and face.

## Discussion

In the medial longitudinal arch, stability is contributed by three metatarsals. The first metatarsal acts as a lever during propulsion along with sesamoids playing an important role in the windlass mechanism. When the first metatarsal is short, the lever arm effect is reduced and the generation of less force for push-off. The plantar aponeurosis is attached to the first three metatarsals, and the thickest part of the plantar fascia is attached to the first metatarsal. In Morton’s foot, during dorsiflexion, great tension is put on the weaker area of fascia attached to the second and third metatarsals [6]. When the windlass mechanism is less effective, the foot goes for pronation in the late stage of propulsion, the push-off comes from the medial side of the hallux. There is shear force across the metatarsals and more force displaced towards the medial side of the hallux. This leads to hallux valgus on the medial side and lesser toes go for more work performance during gripping leading to hammer and claws toes due to tightening of flexor digitorum muscle over usage and development of multiple calluses. In Morton’s foot, the first metatarsal lies behind the third, resembling a chevron [2]. This alters the biomechanics of the foot and bends the foot forward, creating an axis of balance. The transverse axis of the short first metatarsal is angled posteriorly, creating an oblique axis through the first proximal metatarsophalangeal joint, leading to increased friction, pressure, and weight on the forefoot, which in turn leads to metatarsalgia [6-7]. In Morton’s foot, the second metatarsal experiences higher pressure, predisposing it to callus formation and stress fractures [8]. The presence of a short first metatarsal significantly contributes to stress fractures, in the proximal portion of the second metatarsal in 80% of patients [9-10]. An increased load on the second metatarsal results in mid-foot arthrosis in 18.6% of individuals.

Other conditions that mimic Morton's foot include metatarsus primus and metatarsus adductus. Some individuals naturally have a slightly longer second toe and a short first metatarsal, which should not be mistaken for Morton's foot. This misconception can be avoided by carefully measuring the length of all metatarsals, with particular attention to metatarsal protrusion aiding in accurate diagnosis. Errors can be effectively avoided by using the third metatarsal length as a marker for comparison rather than the second. Other than X-ray, the following methods can be used for the diagnosis [2]: (a) The clinician holds the foot with the toes plantar flexed, and a mark is made with a pen in the metatarsophalangeal joint space between the first and third metatarsals to determine the proximity of the first metatarsal with the third metatarsal. (b) Using a Harris mat or ink pad, the patient steps on the ink pad to create a footprint. A point is marked on the heel, and three markings on the first, second, and third metatarsals are connected with lines to the heel. The line to the second metatarsal is taken as a reference, and another line is drawn from it to the metatarsal markings at a right angle. The most distal dot in this configuration is suggestive of Morton's foot. (c) Digital photography method: a 5-inch bent sub-ortholen is used to mark metatarsals and navicular tubercle, and a photo is taken from a height of 40 cm above. Then, a line is drawn from the navicular tubercle to the first and third metatarsals, using the GNU Image Manipulation Program software for calculating metatarsal protrusion. (d) Pressure mapping is an accurate method for understanding the metatarsal response to stress [2]. However, it requires expensive software that is not widely available. The more pressure on the second metatarsal alone does not conclusively indicate Morton’s foot syndrome.

Morton’s extension is used to treat Morton's foot. It is a flexible pad designed to raise the first metatarsal head of about 2-6 mm. In turn, this raises the head of the metatarsal, increases the length of the lever arm of the toe for push-off, lengthens the propulsive phase, and increases the medial longitudinal arch curve. In patients experiencing significant pain and callousing, a metatarsal pad is used to elevate the metatarsal shaft 6-11 mm behind the metatarsal line as it reduces the pressure and alleviates discomfort. Both Morton’s extension and metatarsal pad can be combined and used. Rocker soles may be used to treat metatarsalgia and calluses, diabetic ulcers of the second metatarsal [2,11]. When the second metatarsal is symptomatic and excessively lengthier when compared to the first metatarsal, an osteotomy is done to shorten the second metatarsal. In debilitating Morton's foot syndrome, brachymetatarsal surgery is done to lengthen the first metatarsal [12]. The patient was recommended to use Morton's extension pad to alleviate her symptoms. During the follow-up visit, she expressed a reduction in foot pain compared to previous assessments. In addition, she noted an improvement in her walking posture without strain and observed a moderate decrease in calluses on her foot. There were no cases of Morton’s foot syndrome associated with iron deficiency anemia across the literature, indicating no established correlation. In this patient, the iron deficiency may be attributed to her imbalanced diet and poor socioeconomic status.

## Conclusions

Many patients are unaware that they have Morton's foot, a congenital condition that typically remains asymptomatic. The presence of Morton's foot syndrome is determined by the foot's structure and biomechanics, along with patient symptoms. Despite its estimated prevalence of 4-30% of the population, many cases go undiagnosed. Therefore, it is important to consider Morton's foot in differential diagnosis when observing foot calluses on the feet in individuals with disproportionate toes. Given its genetic component, screening family members is also recommended.
